# Prevalence, Etiology, and Risk Factors of Mastitis in Dairy Cattle in Embu and Kajiado Counties, Kenya

**DOI:** 10.1155/2020/8831172

**Published:** 2020-08-04

**Authors:** Christine M. Mbindyo, George C. Gitao, Charles M. Mulei

**Affiliations:** ^1^University of Nairobi, College of Agriculture and Veterinary Sciences, Department of Veterinary Pathology, Microbiology and Parasitology, P.O. Box 29053-00625, Kangemi, Nairobi, Kenya; ^2^University of Nairobi, College of Agriculture and Veterinary Sciences, Department of Clinical Studies, P.O. Box 29053-00625, Kangemi, Nairobi, Kenya

## Abstract

Bovine mastitis continues to be a leading cause of heavy economic losses in the dairy industry and a public health hazard globally. This cross-sectional study investigated the prevalence, etiologies of clinical and subclinical mastitis, and associated predisposing factors in Embu and Kajiado counties in Kenya. A semistructured questionnaire was administered to 154 smallholder dairy farmers to collect data on management practices, animal factors, and disease history. A total of 395 dairy cows were initially screened for subclinical mastitis using the California mastitis test (CMT), and milk samples were aseptically collected. Both CMT positive and CMT negative samples were analyzed using conventional bacteriological isolation and identification procedures. In the present study, the overall prevalence of mastitis based on CMT and clinical examination was 80% (316/395), out of which 6.8% (27/395) was clinical mastitis, while 73.1% (289/395) was subclinical mastitis. Based on culture, the overall prevalence of clinical and subclinical mastitis was 51.6% (815/1580), 74.4% (294/395), and 76.6% (118/154) at the quarter, cow, and farm level, respectively. From the 1574 milk samples analyzed by cultured, 1016 bacteria were yielded. The predominant bacteria were coagulase-negative *Staphylococcus* (CNS), 42.8% (435/1016), and in decreasing order, *Streptococcus species*, 22.2% (226/1016), *Staphylococcus aureus*, 15.7% (160/1016), and *Pseudomonas aeruginosa*, 5.1% (52/1016), and the least was *Enterobacter* species, 0.7% (7/1016), while 23.7% of the sample yielded no bacterial growth. Risk factor analysis revealed that milking mastitic cows last (*p*=0.002), using a clean udder drying towel for each cow (*p*=0.033) and previous history of mastitis (*p*=0.046) were significantly associated with presence of mastitis. The current study has shown a relatively high prevalence of subclinical mastitis with CNS as predominant bacteria. Therefore, control measures are urgently warranted. Management factors such as milking mastitic cows last, using a clean towel for udder drying for each cow, and culling mastitic cows should be considered and included in the Kenyan mastitis control programs.

## 1. Introduction

Bovine mastitis remains one of the most critical diseases of dairy cows globally [1, 2]. This disease is of particular concern, especially in Africa, including Kenya, where there is limited research on mastitis [3, 4]. Apart from the substantial economic losses associated with the disease, mastitis has serious zoonotic potential and has been associated with the increasing development and the rapid emergence of multidrug resistance strains globally [5–8].

Mastitis, which is the inflammation of the udder and teats, exists in two primary forms: clinical and subclinical mastitis [9, 10]. Clinical mastitis, which is less prevalent, is characterized by systemic signs in the cow and visible abnormalities in the udder and milk [11, 12]. In contrast, subclinical mastitis is more common and results to reduced milk production without observable clinical signs or abnormalities in the udder or milk [13, 14]. For this reason, subclinical mastitis is challenging to diagnose, persists longer in the herd, and is associated with higher losses compared to clinical mastitis [15].

A wide range of microbes have been documented as causative agents of mastitis globally [4, 12]. These include both contagious and environmental bacteria, in addition to fungi, algae, and viruses. Evidence-based studies have shown significant variation in the distribution of mastitis and mastitis-causing pathogens among countries, regions, and farms [16, 17]. These variations are influenced by farm management practices and regional environmental factors [10, 18].

Bacteria are the primary causes of mastitis, and more than 140 different pathogenic species have been reported [4]. Previously, studies had documented major pathogens of mastitis such as *Staphylococcus aureus*, *Streptococcus agalactiae*, and Coliforms [19, 20]. However, current studies by various scholars have reported a change of the causative agents of mastitis from the major pathogens to minor pathogens such as coagulase-negative *Staphylococcus* and other bacilli [14, 21–23]. These reports have shown that these minor pathogens may be playing a significant role in the pathogenesis of mastitis and vary from herd to herd [24, 25]. Hence, this transition warrants a review of traditional control and preventive measures of the disease [23].

Prompt identification and understanding diversity of pathogens associated with mastitis is essential for effective prevention and control [26]. Several studies have reported that subclinical mastitis in the East African region is on the rise. These include studies by Abrahmsén and Persson [15] and discussed elsewhere by [13, 14, 27]. However, studies in Kenya on prevalence and risk factors to mastitis remain limited, while knowledge concerning mastitis pathogens remains scarce [28–30]. Moreover, there has been no comparison of these factors between pastoral and smallholder farming communities. This limitation of studies on mastitis in Kenya is a serious problem because the inability to correctly and accurately identify the pathogen leads to difficulty in selecting the appropriate pathogen-specific treatment or control measure to apply. Moreover, lack of proper identification of the causative agent of mastitis has led to the indiscriminate use of antibiotics, consequently increasing development, and the rapid emergence of multidrug resistance strains [31].

Furthermore, mastitis is of great concern in Kenya, especially in smallholder farms in major towns and environs, such as Kajiado and Embu [1]. In these regions, there is an increasing demand for milk and milk products to cater to the fast-growing human population [32, 33]. Hence, there is an urgent need for extensive research on the status of mastitis and mastitis pathogens in Kenya in order to improve existing control measures and guide treatment. The current study investigated the prevalence, etiology of clinical and subclinical mastitis, and associated risk factors in dairy cows in Embu and Kajiado counties, Kenya.

## 2. Materials and Methods

### 2.1. Ethical Approval

The study was reviewed and approved by the University of Nairobi, Faculty of Veterinary Medicine Biosafety, Animal Use, Care, and Ethics Committee before initiation of the study (FVM/BAUEC/2018/157). To participate in the study, farmers were required to give informed consent verbally.

### 2.2. Study Sites

**Figure 1:**
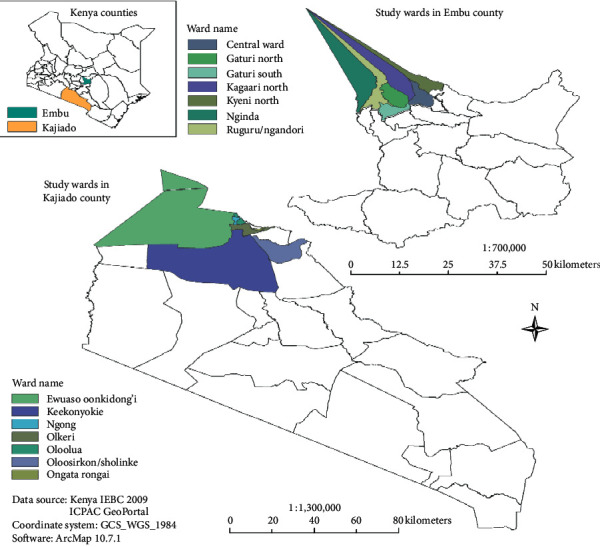
Map of the Embu and Kajiado showing the study sites.

This cross-sectional study was conducted in two counties in Kenya, namely, Embu and Kajiado, between November 2018 and June 2019. In Embu County, the study was conducted in Runyenjes and Kyeni North and in Kajiado County, in Rongai, Ngong, and Kiserian regions (Figure 1).

The study areas broadly fall into two agroclimatic zones. The Kajiado County zone has a hot and humid tropical climate, while Embu is under the trimodal rainy and humid tropical climate. These two counties were purposefully selected based on the high populations of dairy cows in the regions and the increasing demand for cow milk due to the rapidly growing human population in the regions.

Embu County is a high potential area which consists of highlands and lowlands. This county lies between 37.7238°E and 0.6560°S. It rises from about 515 m above sea level at the Tana River basin in the east to over 4570 m above the sea in the northwest which is part of Mt. Kenya. It covers an area of a population of 608,599 persons, most of whom are small-scale farmers. Kajiado County lies between 2.0981°S and 36.7820°E. It covers 21,292.7 km area, and in 2019, the human population was at 1,117,840, and most of whom are traditional pastoralists. The county borders the capital city Nairobi to the west and Tanzania to the south [34].

### 2.3. Study Animals

The study animals were lactating dairy cows of exotic and crosses (mainly crossbreed of exotic and the zebus, a local breed) origin. The cows were in different parities, and stages of lactation from smallholder farmers were randomly selected from the two counties. All cows from Embu were intensively kept, whereas, in Kajiado, animals were semi-intensively and intensively reared.

### 2.4. Study Designs and Sample Size Determination

A cross-sectional design was used in this study. The sample size was calculated using the formula as given by Thrusfield [35]; *n*=(1.96^2^)(*P*_exp_)(1 − *P*_exp_)/*d*^2^ where *n* is the sample size, 1.96 is the *Z* statistic for a level of 95% confidence, *P*_exp_ is expected prevalence, and *d* is the desired absolute precision, which is equal to 5% (0.05). With an expected prevalence of 54.2% [3], a sample size of 381 was obtained; however, 400 animals were sampled instead. A total of 154 available and willing farms owning (<10 cows) were included in the study.

### 2.5. Sampling Technique and Sampling Frame

Initially, Embu and Kajiado counties were purposively selected from the 47 counties in Kenya based on the high population of cows and the increasing demand for milk due to urbanization in these regions. In addition, the counties had different production systems where Kajiado consisted of both semi-intensive and intensive smallholder farmers while Embu consisted of exclusively intensive smallholder farmers. Farms were randomly selected from lists of farmers provided by the veterinary county officers from each of the counties. For a farm to be included in the study, they had to have at least two lactating cows, and the farmer had to be willing to take part in the study. A total of 154 smallholder farms and 400 lactating dairy cows were recruited and sampled between November 2018 and June 2019. In each farm, two cows were randomly selected and sampled. In farms with more than two lactating cows, the remaining cows were randomly sampled, and a maximum of six lactating dairy cows were sampled in such farms. Data for five cows were removed from the study because they were incomplete.

### 2.6. Questionnaire Administration

During the farm visits, data were collected using a pretested semistructured questionnaire administered on each farm via personal interviews. The information collected included farm biodata and farm management practices such as production system (intensive or semi-intensive), housed with a roof (yes or no), floor type (concrete or earthen), bedding (yes or no), cleaning frequency (daily or weekly), proper milking techniques (yes or no), milking mastitic cow last (yes or no), washing of the udder before milking (yes or no), drying of the udder after washing (yes or no), udder towel for each cow (yes or no), routine testing for mastitis (yes or no), use of teat dips (yes or no), dry therapy (yes or no), and culling (yes or no). Cow factors were breed (cross or exotic), stage of lactation recorded in months (1-2) (3–6) (>7), parity (1, 2, 3, 4+), and history of mastitis (yes/no). All collected risk factors were compared to the occurrence of mastitis based on bacteriology results.

### 2.7. California Mastitis Testing and Sample Collection

At the farm, the California mastitis test (CMT) was carried out based on the guidelines described by Schalm [36] and NMC [37]. A total of 400 cows were screened, and milk samples were collected from each quarter. All udders and teats for each cow were physically examined. Detection of clinical mastitis was done by examining the udder and the teats for any inflammation, fever in the animals, and checking the milk's consistency for the presence of clots, blood, and flakes [11]. Following physical examination, screening for subclinical mastitis (SCM) using the California mastitis test (CMT) was done. The CMT results were interpreted subjectively as either negative, trace, 1+, 2+, or 3+, as described by NMC [37]. Based on the CMT, cows were considered positive for SCM if they had readings of (1+, 2+, 3+), whereas negative and trace were taken as negative. The cows were then grouped, and results were recorded as mastitic or nonmastitic. A cow was regarded as mastitis positive if at least one of the quarters was CMT positive. Milk samples were then collected from both CMT positive and negative cows. Briefly, before sampling, the udder was thoroughly washed with water and dried. After disinfecting the teats with 70% ethyl alcohol swabs, followed by stripping 4–5 streams of milk, 5–10 ml of milk was collected from each quarter aseptically and put in separate universal bottles held at a slightly horizontal position to avoid contamination from the udder [11, 37]. The sample bottles were then appropriately sealed and labeled. Samples were refrigerated in ice-boxes with cold packs and transported to the University of Nairobi, Department of Veterinary Pathology, Microbiology, and Parasitology Bacteriology Laboratory, for processing. The samples were cultured immediately or stored in the refrigerator at 4°C for a maximum of a day, awaiting culture.

### 2.8. Bacterial Isolation and Identification

All bacteriological examination was done at the bacteriology laboratory, according to standard methods described in the Laboratory Handbook on Bovine Mastitis [37]. The samples were first brought out of the fridge, left outside to warm up to room temperature (24°C–26°C). Milk samples from five cows were eliminated from the study because they had missing data. Milk samples from 395 were analyzed. Briefly, a 0.01 ml aliquot of each milk sample was aseptically streaked onto the surface of 5% sheep blood agar and MacConkey agar plates (Oxoid, England). The plates were incubated aerobically at 37°C for 18–24 hours. After which, the colony morphology was read and recorded. The plates with no growth were further reincubated for up to 72 hours, after which they were concluded as no growth. Samples yielding more than one colony were grouped as mixed cultures. The distinct colonies were subcultured separately to obtain pure colonies by restreaking. Single colonies from respective isolates were then subcultured onto nutrient agar slants. The slants were stored at 4°C for further use. The bacterial cultures were initially studied morphologically, then microscopically, and later biochemical tests were used to determine the genus and species of pathogens in the sample.

*Staphylococcus* species were identified using growth characteristics, catalase test, tube coagulase testing, and mannitol salt agar (Oxoid, England). *Streptococcus* species were identified using the catalase test and growth characteristics on Edward's media (Oxoid, England) and within the group differentiation was done using the CAMP test. Gram-negative bacteria were identified using colony morphology and lactose fermentation on MacConkey, oxidase test, sugar fermentation, and IMViC tests: I for indole test, M for methyl red test, Vi for Voges–Proskauer test, and C for citrate utilization test (Oxoid England). A cow was considered mastitic positive if at least one quarter was positive for bacterial isolation.

### 2.9. Data Analysis

Data entry and management was done using Microsoft Excel 2016, while data analysis was done using STATA version15. Descriptive statistics were used to calculate the prevalence of clinical and subclinical mastitis. Prevalence was calculated as the proportion of sick over the total population analyzed. Univariate analysis was used to assess the association between the dependent variable (mastitis outcome based on bacterial isolation, 0 = negative, 1 = positive) and independent variables (risk factors). All variables with *p* < 0.05 (5%) were considered for a final model and analyzed using multivariable logistic regression. In this analysis, the statistical significance was set at *p* < 0.05. The output of the model was presented as odds ratio and 95% confidence interval.

## 3. Results

### 3.1. Prevalence of Clinical and Subclinical Mastitis Based on California Mastitis Test and Culture in Study Areas

A total of 1580 quarters from 395 cows in 154 farms from the two counties were analyzed for mastitis in this study. The overall prevalence of mastitis based on CMT and clinical examination was 80% (316/395), of which 6.8% (27/395) were clinically sick and 73.1% (289/395) had subclinical mastitis. Within the counties, Embu reported a higher prevalence in both clinical mastitis and subclinical mastitis, with 8.4% (16/189) and 73.5% (139/189), respectively. Kajiado County reported 5.3% (11/206) in clinical mastitis and 72.8% (150/206) in subclinical mastitis. There was no significant difference between the means of the two counties based on CMT (*p* > 0.3).

Based on the bacterial isolation, the overall prevalence of mastitis in this study, at farm, cow, and quarter level, was 76.6% (118/154), 74.4% (294/395), and 51.5% (815/1580), respectively. Embu County reported higher prevalence at farm and cow level at 78.7% (63/80) and 79.3% (150/189), whereas Kajiado County had 74.3% (55/74) and 69.9% (144/206), respectively. Similarly, Embu County reported a higher prevalence of quarter-level mastitis, 60.1% (455/756), as compared to Kajiado, which had 43.6% (360/824). There was a significant difference between the prevalence of mastitis at cow and quarter level within the two counties (*p* < 0.03; *p* < 0.01).

### 3.2. Prevalence of Causative Bacteria of Mastitis in Embu and Kajiado Counties

Out of the 1580 quarters examined, 6 of them were blind and did not produce any milk. A total of 1574 quarters were cultured for bacterial isolation, which yielded a total of 1016 bacteria. 62% of the samples had a single bacterium, while 37.9% were mixed culture. Overall, from both counties, the most prevalent bacteria were coagulase-negative *Staphylococcus* (CNS) and *Streptococcus* spp.; while *Micrococcus* spp. and *Enterobacter* spp. were the least recovered organisms (Table 1). Also, from the table, *Micrococcus* spp. and *Streptococcus agalactiae* were not recovered from clinically sick quarters. However, on the other hand, all isolated bacteria were reported in subclinical mastitis. In this study, the mastitis-causing organisms frequently identified in the two counties were almost similar. In Embu County, CNS was the most prevalent bacteria isolated in clinical and subclinical mastitis followed by *Streptococcus* spp. and *Staphylococcus aureus*. In Kajiado County, however, *Streptococcus* spp. were the most prevalent bacteria in clinical mastitis, while in subclinical mastitis, CNS was reported highest (Table 1).

In this study, there was a significant difference, in the prevalence of *Bacillus* spp. and *Escherichia coli* recovered between the two counties (*p* < 0.05). Embu County reported a higher prevalence of *Bacillus* spp. and *Escherichia coli* compared to Kajiado. However, the other bacteria isolated from the two counties did not show any statistical significance differences in their prevalence (Table 2).

It is noteworthy that 23.7% (251/1058) of the clinical and CMT positive samples did not yield any bacterial growth.

### 3.3. Mastitis Risk Factors

Risk factors included management factors such as county, production system, housed, floor type, bedding, floor cleaning frequency, proper milking techniques, milking mastitic cows last, washing of the udder before milking, udder drying, udder drying towel for each cow, routine testing for mastitis using CMT, alcohol test, use of teat dips, dry therapy, and culling. Cow factors included stage of lactation, breed, history of mastitis, and parity.  Table 3 represents a summary of the frequency of all variables considered in the study.

### 3.4. Logistic Regression of Risk Factors with the Occurrence of Mastitis

Several risk factors were considered for univariate logistic regression for the presence of mastitis as shown in Table 4. Among the risk factors analyzed, county, bedding, parity, milking mastitic cows last, udder drying towel for each cow, washing hands between milkings, use of teat dips, routine testing for mastitis, and history of mastitis were significantly associated with the presence of mastitis (*p* < 0.05). On the other hand, production system, housed, floor type, proper milking technique, cleaning frequency of the house, floor type, clean udder drying towel, dry cow therapy, culling, breed, and stage of lactation were not significantly associated with mastitis (*p* > 0.05).

All variables with *p* < 0.05 (5%) in the initial univariate regression were included in the multivariable logistic regression analysis. Variables included were county, bedding, milking mastitic cows last, udder drying towel for each cow, washing hands between milkings, teat dips, routine testing for mastitis, parity, and history of mastitis. However, on controlling for confounding effect, parity and bedding were eliminated from the final analysis. The remaining seven variables were analyzed and revealed that milking the mastitic cows last (*p*=0.002), clean udder drying towel for each cow (*p*=0.033), and history of mastitis (*p*=0.046) were significantly associated with the occurrence of mastitis (*p* < 0.05). County, testing for mastitis, use of teat dips, and washing hands between milkings were not statistically significant (*p* > 0.05). Accordingly, the likelihood of occurrence mastitis in dairy cows with a previous history of mastitis was 1.7 higher than in cows, which had no previous history of mastitis (OR = 1.717; 95% CI: 1.061, 2.78). Similarly, there was 2.3 times more likelihood of mastitis occurrence in farms, which did not milk mastitic cows last compared to farms which did (OR = 2.264; 95% CI: 1.645, 4.314). Farms, where each cow did not have its own udder drying towel, were 2.5 more likely to get mastitis than farms where each cow had its own udder towel (OR = 2.491; 95% CI: 1.188,3.154) as shown in Table 5.

## 4. Discussion

Knowledge on prevalence of mastitis, the microbial diversity, and risk factors associated with the disease development would greatly improve prevention and guide on treatment.

In the current study, the overall prevalence of mastitis reported was relatively high (80%). These results were in line with findings in a previous study in Kenya by Ondiek and Kemboi [29], who reported a prevalence of 82.9%. Similar studies by Abebe et al. [2] and Ndahetuye et al. [14] who reported a prevalence of 76% in Ethiopia and 76.2% in Rwanda, respectively, were in close agreement with our findings. However, this study's findings slightly differed with results by Tolosa et al. [38], 85% in Ethiopia, and Abrahmsén and Persson [15], 86.2% in Uganda. On the other hand, these results were higher than reports by Mureithi and Njuguna [28] and Gitau et al. [3], 54.2%, in different parts of Kenya. The inconsistencies in prevalence in these studies could be due to differences in management, environmental, epidemiological, and breed factors [10]. The high prevalence reported in this study could be indicative of inadequate monitoring, control, and prevention measures of mastitis in the study areas.

The present study reported clinical mastitis of 6.8%. These findings were closely related to what was reported by Sarba and Tola [39], 9.9%, and Zeryehun and Abera [13], 10%, in Ethiopia. However, they were lower than findings by Amer et al. [18] in Japan, who reported a prevalence of 12%, Tolosa et al. [38] and Mekibib et al. [40] in Ethiopia, and Levison et al. [41] in Canada, who reported prevalence of 12%, 11%, 22.7%, and 23%, respectively. In contrast, our results were higher than the prevalence reported by Gitau et al. [3] and Gao et al. [17], who reported prevalence of 0.5 and 0.9% in a different part of Kenya, and 3.3% in China, respectively.

Subclinical mastitis in this study was about 74%. These findings were in agreement with studies by Abebe et al. [2], 76% in Ethiopia, and Ndahetuye et al. [14], 76.2% in Rwanda. Our findings were higher than the results reported by Mureithi and Njuguna [28], 64% in Thika, Kenya, and Gitau et al. [3], 49.6% and 58.7%, in Nyeri and Nakuru, Kenya, and as reported elsewhere by [21, 42]. However, this study's findings were lower than the findings by Ondiek and Kemboi [29], 82.9%, Egerton, Kenya, and Tolosa et al. [38], 85% in Ethiopia. Previous studies have shown that mastitis differs from one country to country and between farms and cows [4]. This remarkable variability in prevalence in clinical and subclinical mastitis could be attributed to ineffective mastitis control programs, environmental factors, and poor hygiene standards in the study areas [15, 16, 42, 43].

This study reported a higher prevalence of subclinical mastitis (74%) than clinical (7%) mastitis. These findings agree with other studies by Zeryehun and Abera [13] and as reported elsewhere by [3, 39]. These significant differences could be attributed to the fact that clinical mastitis can easily be diagnosed and treated [4], whereas, on the other hand, the subclinical form has no physical abnormalities; hence, it is hardly diagnosed by the farmers and continues to be a source of infection in the farm [2, 43]. In addition, ineffective mastitis control programs and poor hygiene standards in the study areas could have been a key contributor [15]. Therefore, there is a need for implementation of continued mastitis monitoring and control programs in the study areas such as the creation of awareness on subclinical mastitis and possible risk factors.

In the present study, Embu County had a significantly higher cow and quarter-level prevalence of mastitis compared to Kajiado. This high prevalence in Embu County may be associated with poor hygiene and the wet weather experienced (March–June 2019) during the sampling period. Similar findings were found in Bangladesh, where higher prevalence of mastitis was reported during wet seasons [44]. Moreover, in Embu County, cows stayed in dirty bedding and poorly drained houses. FAO [1] reported that poor drainage and dirty beddings were associated with high contamination with environmental mastitis pathogens. The authors recommend urgency to implement hygiene measures for successful control of mastitis in the region.

The current study reported coagulase-negative *Staphylococcus* (CNS) as the most predominant bacteria in both clinical and subclinical mastitis. These findings agree with studies from different countries globally, which have reported CNS as emerging bacteria of mastitis [14, 16, 18, 23, 45, 46]. However, the results contrasted with finding by Gitau et al. [3], Mureithi and Njuguna, [28], and Ondiek and Kemboi [29], all in Kenya who reported *Staphylococcus aureus* as the dominant mastitis pathogen. The present results show that CNS, which was initially classified as a minor pathogen of mastitis, is emerging as an important mastitis pathogen in the Kenyan dairy cows. This transition from *Staphylococcus aureus* to CNS may imply the need to revise control procedures. The high prevalence of CNS in this study might be explained by the fact that the bacterium which is a normal flora of the skin, could be originating from milker's hands or cow skin during milking [23, 25]. Also, studies have shown that CNS can originate from the cow's environment [22, 47]. However, further studies will be necessary for Kenya to investigate the epidemiology and the specific pathogenic species involved in CNS mastitis.

Streptococcal mastitis reported in this study was 22.2%. These findings were comparable to studies by Birhanu et al. [48], Gitau et al. [3], and Kalmus et al. [49], who reported a prevalence of 22%, 20.6%, and 18% respectively. However, the findings were lower than reports by Vakkamäki [24], Amer et al. [18], Ndahetuye et al. [15], and Mekibib et al. [40] who reported prevalence of 14%, 9%, 1.4%, and 5.5% respectively. This high prevalence of environmental *Streptococcus* in this study could possibly be due to udder contamination from the cow's environment or the water source [18].

Furthermore, *Bacillus* species was reported in this study at a prevalence of 7.6%. To the best of our knowledge, this was the first time *Bacillu*s spp. was isolated in mastitis in Kenyan dairy. Other studies have reported that *Bacillus* spp. as an important pathogen of mastitis [50, 51]. Our findings sharply contrasted with what was reported by Amer et al. [18] in Japan, 22%. However, our results were higher than by Mekibib et al. [40], Zeryehun and Abera [13], and Fisseha et al. [52], in Ethiopia who reported prevalence of 1.3%, 2.7%, and 4.2%, respectively. The presence of *Bacillus* spp. in our findings could be due to environmental contamination of the udder by mud or manure [50, 51]. Therefore, improving environmental hygiene, milking hygiene, and the use of teat sealants could reduce infections [53].

In this study, the prevalence of *Bacillus* spp. and *Escherichia coli* was significantly higher in Embu County compared to Kajiado. Studies elsewhere have shown that such differences are commonly linked to geographic variations [26, 54].

No growth was reported in 23.7% of the clinical and subclinical mastitis cases. Similar results were reported by Levison et al. [41], Canada, 23%. However, these findings were lower than what was reported by Richards [30], 37.5% in a recent study in Kenya, but higher than reported by Gitau et al. [3], 10.4% in Kenya. These findings could be due to limitations of the culture methods, low level of bacteria in milk, cow pretreated with antibiotics, and causative agents of mastitis not bacteria [55].

This study revealed that farms which did not milk mastitic cows last were more likely to have mastitis than the farms where mastitic cows were milked last. These findings were in agreement with the reports by Abebe et al. [2], in Ethiopia, and Nielsen and Emanuelson [56], in Sweden. They reported that failure to milk mastitic cows last increased the spread of mastitis in farms from one cow to another during milking. These findings may explain the reason for high farm-level prevalence in this study since all farmers used hand milking. Farmers need to be educated on the importance of knowing the cow's udder health status and encouraged to milk mastitic cows last to prevent the spread of mastitis [56].

In this study, cows with a previous history of mastitis were more likely to have mastitis again compared to cows with no previous history of mastitis. A similar finding was reported by Mekonnen et al. [57] in Ethiopia, Kumar et al. [58] in India, and Oliveira et al. [59] in Brazil. However, it contrasted with findings by Abebe et al. [2], Ethiopia, who did not find any association between history of mastitis and future occurrence of mastitis. In our study, inadequate screening and treatment of subclinical mastitis, lack of correct, and specific identification of the mastitis pathogens in clinical cases may have led to the recurrence of mastitis. In addition, indiscriminate use of antibiotics by farmers leading to the development of mastitis resistance pathogens was believed to be a critical contributor for the recurrence of mastitis in this current study (unpublished data). Certainly, such cows need to be culled to prevent further transmission of mastitis in the farms [12, 58].

Farms that did not use an udder drying towel for each cow had significantly higher mastitis than farms that used a drying towel for each cow. This agrees with finding by Abebe et al. [2] and Mekonnen et al. [57] both from Ethiopia who reported that the use of the same drying towel for the herd was responsible for spreading mastitis pathogens. This may explain the high prevalence of *Staphylococcus* species reported in this study. Since these organisms are part of the normal flora of the udder and the teats, they can easily be spread through the use of the same drying towel during milking [22, 47].

## 5. Conclusion

The current study reported a high prevalence of subclinical mastitis. Coagulase-negative *Staphylococcus* (CNS) was the predominant mastitis pathogen. Based on the high prevalence of mastitis and CNS, we advise routine monitoring of this emerging pathogens of mastitis, and control measures should be applied in the affected farms. In addition, this study reported some key mastitis risk factors such as previous history of mastitis, using a clean udder drying towel for each cow, and milking mastitic cows last. These factors should be adopted in mastitis control programs in the regions.

## 6. Recommendation

Further studies need to be undertaken to determine the epidemiology of CNS mastitis and circulating species in the study regions. This study was carried out during a rainy season; further studies should be done to assess the role of seasonality in the occurrence of mastitis in Kenya.

## Figures and Tables

**Table 1:** Summary table of the distribution of bacteria isolated from clinical and subclinical mastitis in Embu and Kajiado counties, Kenya.

Bacteria	Clinical mastitis (%)		Subclinical mastitis (%)		Total (%)
	Embu County	Kajiado County	Embu County	Kajiado County	Combined
Coagulase-negative *Staphylococcus*	45.4 (20/44)	22.2 (4/18)	41.4 (227/547)	45.2 (184/407)	42.8 (435/1016)
*Streptococcus* spp.	22.7 (10/44)	38.8 (7/18)	20.1 (110/547)	24.3 (99/407)	22.2 (226/1016)
*Streptococcus agalactiae*	—	—	0.1 (1/547)	0.7 (3/407)	0.4 (4/1016)
*Staphylococcus aureus*	13.6 (6/44)	22.2 (4/18)	13.8 (76/547)	18.1 (74/407)	15.7 (160/1016)
*Bacillus* spp.	9 (4/44)	—	10.6 (58/547)	3.6 (15/407)	7.6 (77/1016)
*Pseudomonas aeruginosa*	2.2 (1/44)	—	5.3 (29/547)	5.4 (22/407)	5.1 (52/1016)
*Escherichia coli*	6.8 (3/44)	5.5 (1/18)	4.2 (23/547)	1.2 (5/407)	3.1 (32/1016)
*Klebsiella* spp.	—	5.5 (1/18)	2 (11/547)	0.4 (2/407)	1.4 (14/1016)
*Micrococcus* spp.	—	—	1 (6/547)	0.7 (3/407)	0.9 (9/1016)
*Enterobacter* spp.	—	5.5 (1/18)	1 (6/547)	—	0.7 (7/1016)
Total	4.3 (44/1016)	1.7 (18/1016)	53.8 (547/1016	40 (407/1016)	100 (1016/1016)

**Table 2:** Effect of counties on the prevalence of mastitis pathogens.

Bacteria	Embu (%) *n* = 591	Kajiado (%) *n* = 425	Total (%) *n* = 1016
Coagulase-negative *Staphylococcus*	41.7^a^ (247/591)	44.2^a^ (188/425)	42.8 (435/1016)
*Streptococcus* spp.	20.3^a^ (120/591)	24.9^a^ (106/425)	22.2 (226/1016)
*Streptococcus agalactiae*	0.1^a^ (1/591)	0.7^a^ (3/425)	0.4 (4/1016)
*Staphylococcus aureus*	13.8^a^ (82/591)	18.3^a^ (78/425)	15.7 (160/1016)
*Bacillus* spp.	10.4^a^ (62/591)	3.5^b^ (15/425)	7.6 (77/1016)
*Pseudomonas aeruginosa*	5^a^ (30/591)	5.5^a^ (22/425)	5.1 (52/1016)
*Escherichia coli*	4.3^a^ (26/591)	1.4^b^ (6/425)	3.1 (32/1016)
*Klebsiella* spp.	1.8^a^ (11/591)	0.7^a^ (3/425)	1.4 (14/1016)
*Micrococcus* spp.	1^a^ (6/591)	0.7^a^ (3/425)	0.9 (9/1016)
*Enterobacter* spp.	1^a^ (6/591)	0.2^a^ (1/425)	0.7 (7/1016)

*n* = total number of bacterial isolates. Different superscript letters on the same row denote a subset of county category whose proportions differ significantly within the row (*p* < 0.05). Similar superscript letters on the same row denote a subset of county category whose proportions do not differ significantly within the row (*p* > 0.05).

**Table 3:** Distribution of farm management practices and cow factors associated with mastitis in 395 dairy cows in Kajiado and Embu counties.

Variable	Group description	Mastitis positive cases (%)
(1) County	Kajiado (*n* = 206)	144 (49)
	Embu (*n* = 189)	150 (51)
(2) Production system	Semi-intensive (*n* = 73)	48 (16.3)
	Intensive (*n* = 322)	246 (83.7)
(3) Housed with a roof	Yes (*n* = 388)	290 (98.6)
	No (*n* = 7)	4 (1.4)
(4) Floor type	Concrete (*n* = 305)	224 (76.2)
	Earthen (*n* = 90)	70 (23.8)
(5) Bedding material	Yes (*n* = 123)	83 (28.2)
	No (*n* = 272)	211 (71.8)
(6) Cleaning of floor	Daily (*n* = 298)	216 (73.5)
	Weekly (*n* = 97)	78 (26.5)
(7) Milking the cows last	Yes (107)	64 (21.8)
	No (288)	230 (78.2)
(8) Proper milking technique	Yes (387)	7 (2.4)
	No (8)	287 (97.6)
(9) Use of clean udder drying towel	Yes (375)	278 (94.6)
	No (20)	16 (5.4)
(10) Use of clean udder drying towel for each cow	Yes (*n* = 165)	106 (36.1)
	No (*n* = 230)	188 (63.9)
(11) Washing hands between milkings	Yes (*n* = 171)	113 (38.4)
	No (*n* = 224)	181 (61.6)
(12) Use of teat dips	Yes (*n* = 52)	31 (10.5)
	No (343)	263 (89.5)
(13) Dry therapy	Yes (94)	66 (22.4)
	No (301)	228 (77.6)
(14) Culling	Yes (*n* = 77)	55 (18.7)
	No (*n* = 318)	239 (81.3)
(15) Test for mastitis	Yes (249)	177 (60.2)
	No (146)	117 (39.8)
(16) Breed	Cross (*n* = 26)	16 (5.4)
	Exotic (*n* = 369)	278 (94.6)
(17) Parity	1 (*n* = 102)	68 (23.1)
	2 (*n* = 99)	75 (25.5)
	3 (*n* = 94)	72 (24.5)
	>4 (*n* = 100)	79 (26.9)
(18) Stage of lactation	Early (1-2 months) (*n* = 106)	85 (28.9)
	Mid (3–6 months) (*n* = 134)	94 (32)
	Late > 7 months (*n* = 155)	115 (39.1)
(19) History of mastitis	Yes (*n* = 237)	167 (56.8)
	No (*n* = 158)	127 (43.2)

**Table 4:** Univariate logistic regression analysis between different risk factors and cow-level mastitis (defined as culture positive) in 395 dairy cows in Embu and Kajiado counties.

Variable	Number examined, *n* = 395	Mastitis culture results (%)		95% CI		*p* value
		Negative (%)	Positive (%)	Lower	Upper	
County						
Kajiado	206	62 (30.1)	144 (69.9)			
Embu	189	39 (20.6)	150 (79.4)	1.044	2.626	0.032^*∗*^
Production system						
Semi-intensive	73	25 (34.2)	48 (65.8)			
Intensive	322	76 (23.6)	246 (76.4)	0.975	2.915	0.062
Housed						
No	7	3 (42.9)	4 (57.1)			
Yes	388	98 (25.3)	290 (74.7)	0.488	10.091	0.302
Floor type						
Concrete	305	81 (26.6)	224 (73.4)			
Earthen	90	20 (22.2)	70 (77.8)	0.724	2.212	0.408
Bedding						
Yes	123	40 (32.5)	83 (67.5)			
No	272	61 (22.4)	211 (77.6)	1.039	2.675	0.034^*∗*^
Cleaning of floors						
Daily	298	82 (27.5)	216 (72.5)			
Weekly	97	19 (19.6)	78 (80.4)	0.888	2.734	0.122
Milking the cows last						
Yes	107	43 (40.2)	64 (59.8)			
No	288	58 (20.1)	230 (79.9)	1.645	4.314	0.001^*∗*^
Proper milking technique						
Yes	387	100 (25.8)	287 (74.2)			
No	8	1 (12.5)	7 (87.5)	0.296	20.07	0.407
Drying towel						
Yes	375	97 (25.9)	278 (74.1)			
No	20	4 (20)	16 (80)	0.455	4.277	0.560
Towel for each cow						
Yes	165	59 (35.8)	106 (64.2)			
No	230	42 (18.3)	188 (81.7)	1.570	3.954	0.001^*∗*^
Washing hands between milkings						
Yes	171	58 (33.9)	113 (66.1)			
No	224	43 (19.2)	181 (80.8)	1.365	3.419	0.001^*∗*^
Teat dips						
Yes	52	21 (40.4)	31 (59.6)			
No	343	80 (23.3)	263 (76.7)	1.213	4.09	0.01^*∗*^
Dry therapy						
Yes	94	28 (29.8)	66 (70.2)			
No	301	73 (24.3)	228 (75.7)	0.792	2.217	0.284
Culling						
Yes	77	22 (28.6)	55 (71.4)			
No	318	79 (24.8)	239 (75.2)	0.694	2.11	0.501
Test for mastitis						
No	249	72 (28.9)	177 (71.1)			
Yes	146	29 (19.9)	117 (80.1)	1.005	2.68	0.048^*∗*^
Breed						
Crosses	26	10 (38.5)	16 (61.5)			
Exotic	369	91 (24.7)	278 (75.3)	0.230	1.195	0.124
Parity						
1	102	34 (33.3)	68 (66.7)			
2	99	24 (24.2)	75 (75.8)	0.843	2.896	0.156
3	94	22 (23.4)	72 (76.6)	0.401	3.074	0.126
4+	100	21 (21)	79 (79)	0.416	3.543	0.05^*∗*^
Stage of lactation						
Early	106	21 (19.8)	85 (80.2)			
Mid	134	40 (29.9)	94 (70.1)	1	2.050	0.444
Late	155	40 (25.8)	115 (74.2)	1.223	3.152	0.078
History of mastitis						
No	237	70 (29.5)	167 (70.5)			
Yes	158	31 (19.6)	127 (80.4)	1.061	2.78	0.028^*∗*^

^*∗*^Factors significant at *p* < 0.05; CI: confidence interval.

**Table 5:** Multivariable logistic regression analysis between different risk factors and cow-level mastitis (defined as culture positive) in 395 dairy cows in Embu and Kajiado counties.

Variable	Category (yes/no)	OR	95% CI		*p* value
			Lower	Upper	
Milking mastitic cows last	Yes	1			
	No	2.264	1.645	4.314	0.002^*∗*^
Towel for each cow	Yes	1			
	No	2.491	1.57	3.954	0.033^*∗*^
Use of teat dips	Yes	1			
	No	2.227	1.213	4.09	0.368
History of mastitis	No	1			
	Yes	1.717	1.061	2.78	0.046^*∗*^
Test for mastitis	No	1			
	Yes	1.641	1.005	2.68	0.11
Washing hands between milkings	Yes	1			
	No	2.161	1.365	3.419	0.641
County	Kajiado	1			
	Embu	1.656	1.044	2.626	0.817

^*∗*^Factors statistically significant at *p* ≤ 0.05; OR = odds ratio; CI: confidence interval.

## Data Availability

Data associated with this research article are available upon request to the corresponding author.

## Supplementary Materials

Supplementary Materials A questionnaire (submitted as a supplementary file) was used to evaluate the risk factors associated with occurrence of mastitis in dairy cows, in Embu and Kajiado counties, Kenya.
