# Supplementary material for: Prevalence, Etiology, and Risk Factors of Mastitis in Dairy Cattle in Embu and Kajiado Counties, Kenya
Source: Vet Med Int. 2020 Aug 4;2020:8831172. doi: 10.1155/2020/8831172 (PMC7424489; doi:10.1155/2020/8831172)
Supplement: Supplementary Materials — A questionnaire (submitted as a supplementary file) was used to evaluate the risk factors associated with occurrence of mastitis in dairy cows, in Embu and Kajiado counties, Kenya. [file 8831172.f1.pdf]

Prevalence, Etiology and Risk Factors of Mastitis in Dairy Cattle in Embu and Kajiado Counties, Kenya

FARMS SURVEY QUESTIONNAIRE FOR DAIRY FARMERS

To be filled once for each farm

**Demographic information**	
Farm no	
Name of the respondent	
Gender	
Telephone Number of respondent	
Age	
Date of the interview	
County	
Sub –County	
Division	
Location	
Village	
GPS coordinates	
Agro-ecological zone (AEZ)	

Farm Background

1. Who manages the farm?

   A) Owner B) Relative C) Employee

2. What is your level of education?

   A) Informal __B) Primary___C) Secondary ______ D) University _____

3. For how long have you been keeping dairy cattle

   A) <1 year _____ b)>1 year and <5 years ______ c) >5 year ______

4. Production system

   A) Intensive ___ B) Semi intensive____ C) Extensive____

5. How many dairy cows do you have? ____
6. What cattle breed do you keep a) Local ____ b) Exotic____ c) Crosses___
7. Do you keep records? Yes _____ No______

   If yes specify a) Production____ b) Breeding____ C) others (specify)

8. Any given to the cows' supplements? a) No ____ b) Yes ____
9. Are the cattle housed with a roof? A) No ____ B) Yes ____
10. If housed, what is the nature of the floor where the milking cows lie down? A) Concrete____ B) Earthen ___
11. Presence of Bedding Yes ____ No ____
12. How often is the floor cleaned?

    A. Daily____ B) Weekly____C) Monthly____ D) Others (specify)____

Mastitis and control practices

1. Have you ever experience cases of mastitis in the farm? 1)Yes __ 2) No ____
2. Do you milk mastitic cows last? 1)Yes 2) No
3. Is the cow milked using proper technique (observe)? 1) Yes 2) No
4. Do you wash hands before milking the cows? 1)Yes ______ 2) No _______
5. a) Do you wash the udder pre-milking? 1)Yes 2) No

    b) Is the udder dried before milking with clean cloth/towel/paper? 1)Yes 2) No

    c) Is a different clean drying cloth/towel/paper used for each milking cow? 1) Yes __ 2) No ____

    d) if you have > 1 milking cow, do you wash your hands between milking cows? Yes __ No _

6. Do you use a teat dip post milking? 1) Yes ___ 2) No ____

7. Do you practice dry cow therapy? 1) Yes __ 2) No ____

8. Do you cull chronically infected cows? 1) Yes ____2) No ____

9. Who treats your animals?

    a) Vet Surgeon b) Animal Health Assistant c) Self d) Others (specify)

10.Do they perform any test to confirm mastitis?

a) CMT b) Alcohol test c) Culture d) Other (specify)

11. For how long did mastitis during the last case take to resolve (in general)?

    a) One week

    b) Above one week

    c) Never resolved

Cow characteristics details

**Cow ID**	**Breed**	**Parity**	**Milk production per day in L**	**Stage of lactation (1-2Early (3-6) mid (>7)late**	**History of mastitis(yes/No)**	**CMT results for each quarter**
Cow 1						FR<br>HR<br>FL<br>HL
Cow 2						FR<br>HR<br>FL<br>HL
